
JOURNAL INFORMATION
==============================
NLM Title Abbreviation: J Clin Transl Sci
Journal ID: J Clin Transl Sci
NLM Journal ID: 101689953
Journal ID: CTS

Journal of Clinical and Translational Science

EISSN: 2059-8661
Publisher: Cambridge University Press

ARTICLE INFORMATION
==============================
PMCID: PMC6799171
DOI: 10.1017/cts.2018.26
Article ID: S2059866118000262
Article ID: 00026
Article version: 1
Subjects: Back Cover (IBC, OBC) and matter

CTS volume 1 issue S1 Cover and Back matter

Publication date: 2017 Sep
Electronic publication date: 2018 May 10
Volume: 1
Issue: Suppl 1
First page: b1
Last page: b1
Copyright: © The Association for Clinical and Translational Science 2018
Copyright year: 2018
Copyright holder: The Association for Clinical and Translational Science
License: This is an Open Access article, distributed under the terms of the Creative Commons Attribution licence (http://creativecommons.org/licenses/by/4.0/), which permits unrestricted reuse, distribution, and reproduction in any medium, provided the original work is properly cited.
License URL: https://creativecommons.org/licenses/by/4.0/

Figure count: 0
Table count: 0
Page count: 1

==============================
Journal of Clinical and Translational Science

ACTS
ASSOCIATION FOR CLINICAL AND TRANSLATIONAL SCIENCE

© The Association for Clinical and Translational Science 2018. This is an Open Access article, distributed under the terms of the Creative Commons Attribution licence (http://creativecommons.org/licenses/by/4.0/), which permits unrestricted reuse, distribution, and reproduction in any medium, provided the original work is properly cited.
